# First report of the isolation of *Balamuthia mandrillaris* in the northern region of Japan

**DOI:** 10.1007/s00436-018-5980-x

**Published:** 2018-06-30

**Authors:** Kanako Yamanouchi, Hiroaki Arima, Yamato Sakamoto, Kazuki Kanto, Kosuke Kasai, Koichi Ito, Takashi Inaba

**Affiliations:** 1 Department of Bioscience and Laboratory Medicine, Hirosaki University Graduate School of Health Sciences, 66-1 Hon-cho, Hirosaki, 036-8564 Japan; 2 Department of Medical Technology, Hirosaki University School of Health Sciences, 66-1 Hon-cho, Hirosaki, 036-8564 Japan

**Keywords:** Free-living amoeba, *Balamuthia mandrillaris*, Soil, *Balamuthia* 16S rRNA gene, Japan

## Abstract

*Balamuthia mandrillaris* is a free-living amoeba that lives in soil and water near human settlements. *B. mandrillaris* was first isolated from a mandrill baboon that died at the San Diego Zoo Wildlife Park in California in 1986, and the first human infection was reported in 1990. Although reported *B. mandrillaris* infections are often not properly characterized, it appears that *B. mandrillaris* invades the living body from the soil and water, either via a wound or the nasal cavity. Most confirmed infections have originated in South and North America. *B. mandrillaris* inhabits warm climates and is recognized as a pathogen in warm areas such as desert climates and tropical climates. *B. mandrillaris* has been isolated from environmental samples since 2000, most of which originated from warm areas such as step climates, tropical climates, and desert climates. However, *B. mandrillaris* may survive in diverse environments, although fewer granulomatous amebic encephalitis patients have been reported in colder Japanese and Northern European regions. In the present study, we conducted a survey of 13 soil samples in Aomori Prefecture located at the northernmost tip of Japan Honshu and successfully isolated one strain of *B. mandrillaris* from soil for the first time in Japan. In addition, *B. mandrillaris* gene was detected from several soils. This confirms that *B. mandrillaris* is capable of spreading to a wider climatic region.

## Introduction

Protozoan amoebae are classified according to their morphological and genetic features, and inhabit distinct niches in the environment, such as soil, seawater, and indoor dust (Schuster and Visvesvara 2004; Rodríguez-Zaragoza 2008). Among the many genera of free-living amoebae, only four have an association with human disease: *Acanthamoeba* spp., *Balamuthia mandrillaris*, *Naegleria fowleri*, and *Sappinia diploidea*. These are termed amphizoic amoebae because they live both as parasites and in the natural environment. Free-living-amoebae can infect mammals, including humans, and cause granulomatous amebic encephalitis (GAE), skin amebiasis, and amebic keratitis (AK) (Szenasi et al. 1998; Schuster and Visvesvara 2004; Tsvetkova et al. 2004). Among these pathogenic amoebae, *B. mandrillaris* is the most recently discovered and is known to have high lethality, with over 90% of cases leading to death. *B. mandrillaris* was first discovered in a mandrill baboon (*Papio sphinx*) that died of encephalitis at the San Diego Zoo Wildlife Park in California in 1986. Subsequently, more than 200 human infections have been reported worldwide up to 2008 (Visvesvara et al. 1990; Siddiqui and Khan 2008).

Nine cases of *Balamuthia* infection have been confirmed in Japan (Table 1), and we confirmed an infection as recently as 2014 (Kenji 2010; Itoh et al. 2015). Among these domestic cases, healthy individuals without other immunological abnormalities have been identified, indicating a high risk of a generalized infection. In the present study, we isolated *B. mandrillaris* by cultivating soil collected in the Aomori Prefecture, which is at the northernmost tip of Honshu Island, in order to clarify the habitat of *B. mandrillaris* in the natural environment in Japan. Culturing *B. mandrillaris* from the natural environment is complicated and difficult (Niyyati et al. 2016), and we therefore attempted to detect the *Balamuthia* 16S rRNA gene in DNA extracted from soil samples.

**Table 1:** The occurrence of free-living amoeba infection in Japan

Case number	Site	Age/sex	Year	Source	History, notices	Reference
1	Tokyo pref.	27/F	1976	*Acanthamoeba* spp.	Trip to the mountains	Nakamura et al. 1979
2	Yamagata pref.	56/M	1986	*B. mandrillaris*	Diabetes, cellulitis of left upper limb	Uenohara et al. 1987
3	Okayama pref.	78/F	1989	*B. mandrillaris*	Mucocutaneous-ocular syndrome	Monobe et al. 1991
4	Miyazaki pref.	72/F	1995	*B. mandrillaris*	HTLV-1 carriers	Hayashi et al. 1996
5	Niigata pref.	53/M	1995	*B. mandrillaris*	Stomach, nephrectomy due to stomach cancer	Hayashi et al. 1997
6	Saga pref.	25/F	1996	*N. fowleri*	No travel history, no contact with water	Sugita et al. 1999.
7	Miyazaki pref.	51/F	2006	*B. mandrillaris*	Epilepsy	Yamasaki et al. 2011
8	Aichi pref.	56/F	2010	*B. mandrillaris*	No history	Kenji 2010
9	Tokushima pref.	68/M	2012	*B. mandrillaris*	Hepatitis C	Bando et al. 2012
10	Aichi pref.	72/F	unknown	*B. mandrillaris*	Hypertension	Kato et al. 2013
11	Kyoto pref.	81/M	2014	*B. mandrillaris*	Sjogren’s syndrome	Itoh et al. 2015

National Institute of Infectious Diseases: IASR (Vol. 31 p. 334–335: November 2010), additional notes

## Materials and methods

### Collection of soil samples

**Fig. 1:**
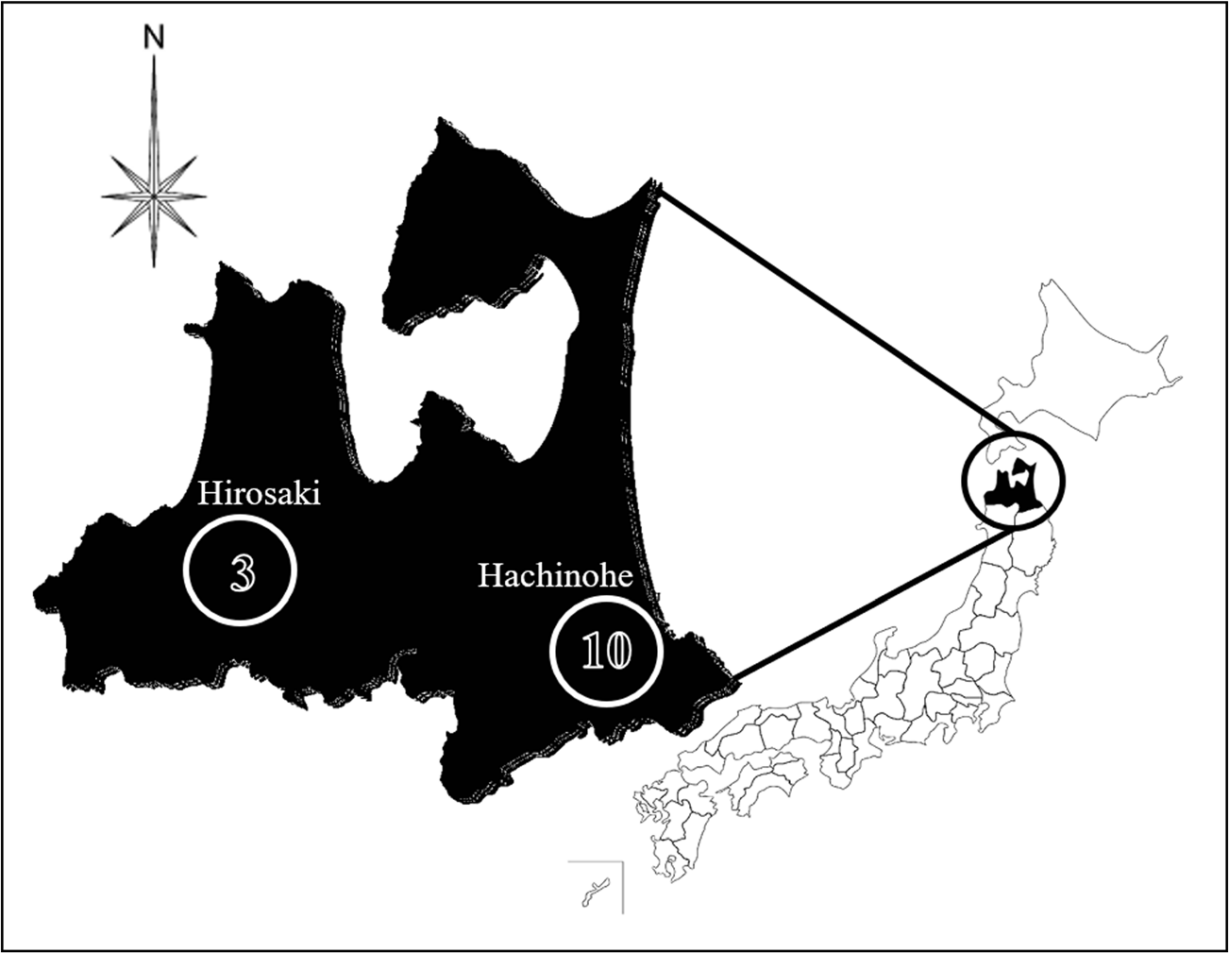
Soil sampling place in Japan

Surface soil was collected at a depth of about 10 cm after removing leaf debris. Ten samples were collected in Hachinohe city located on the southern part of the Pacific side, and three samples in Hirosaki located on the inland western part of Aomori Prefecture (Fig. 1). Soil was collected in general households, agricultural lands, university premises, and a shrine (Table 2).

**Table 2:** Soil sampling place

No.	Area	Site for samples collecting
1	Hachinohe	Horse ranch
2		Farm
3		Farm
4		Bamboo grove
5		Compost garbage disposal soil
6		General household garden
7		Paddy field
8		Farm
9		Farm
10		Farm
11	Hirosaki	Playing field
12		Playing field side
13		Shrine

The collected soil was cultured prior to extraction of DNA for *Balamuthia*-specific PCR. Soil samples were stored in a refrigerator at 4 °C and used for culture and DNA extraction within 3 months of collection.

### Isolation of amoebae by soil culture

*Acanthamoeba* type II (MK strain) from our laboratory collection was heat treated (heat-treated *Acanthamoeba* MK: HTAM) and spread on 1.5% non-nutrient (NN) agar for cultivation. *Acanthamoeba* MK strain used for preparation of HTAM was cultured in PYG medium to a confluent state. Thereafter, *Acanthamoeba* MK strain washed twice with pH 7.2 phosphate-buffered saline (PBS(−)), adjusted to 3000/μl and heat treated by autoclaving at 121 °C for 15 min. Heat-treated *Acanthamoeba* MK strain was cultured in PYG medium and confirmed dead. Soil (10 g) was suspended in 10 ml of KCM medium (7 mg/l KCl, 8 mg/l CaCl_2_, and 8 mg/l MgSO_4_•7H_2_O), and large soil particles were removed using gauze filtration. The filtrate was centrifuged at 800*g* for 5 min to obtain sediment. The sediment was applied at the center of a petri dish containing 1.5% NN agar medium and cultured at 30 °C for 10 days. Each culture was inspected once daily. When large dendritic amoebae were observed, the colony was excised and transplanted on fresh agar. The large amoebae were subsequently cultured in liquid Balamuthia medium BM-3 (Schuster and Visvesvara 1996) and soil solution (SS) medium.

SS medium was prepared by adding 50 g of soil to 1000 ml of physiological saline. The soil suspension was centrifuged at 800*g* for 5 min, and the supernatant was recovered and sterilized by filter sterilization with a 0.22-μm filter (Sartolab, Germany) as the SS medium. The soil type used to prepare SS medium has previously been used to ascertain the habitat of large amoebae.

### DNA extraction from soil

Soil (10 g) was weighed in a conical tube, and 4 g of 350 μmΦ glass beads and 400 mg of skim milk were added. This was followed by the addition of 4 ml of sodium dodecyl sulfate (SDS) lysis buffer [0.5 M Tris HCl(pH 8.0), 0.1 M NaCl, 10% SDS, filter sterilized after creation] and 10 ml of pH 8.0 PBS (−), and the mixture was then vigorously stirred at 1300*g* for 15 min. After stirring, the mixture was heated at 60 °C for 5 min and then stirred at 1300*g* for 15 min. The soil mixture was centrifuged at 2300*g* for 10 min, and the supernatant was recovered. An equal volume of phenol:chloroform:isoamyl alcohol (25:24:1, PCI; NIPPON GENE, Japan) was added to the supernatant, and the mixture was vigorously stirred and then centrifuged at 9000*g* for 20 min. After recovering the supernatant, isopropanol and 3 M sodium citrate were added, and the mixture was incubated at − 30 °C for 30 min, and then centrifuged at 13,000*g* for 20 min to obtain the sediment. The sediment was dissolved in 600 μl of distilled water and finally purified using an Isoil DNA extraction kit (NIPPON GENE, Japan).

### DNA extraction of isolated amoeba

Large amoeba subcultured on 1.5% NN agar were recovered in KCM medium and then centrifuged at 800*g* for 5 min for DNA purification using a QIAamp DNA Mini Kit (QIAGEN, USA).

### *Balamuthia*-specific polymerase chain reaction and sequencing

The purified DNA was adjusted to 10~200 ng/μl, and PCR was carried out using with Buffer II (Thermo Fisher Scientific, USA). The PCR primer was 5’Balspec 16S (5′-CGCATGTATGAAAGAAGACCA-3′) and Balspec 16Sr 610 (5′-CCCCTTTTTAACTCTAGTCATATAGT-3′), with an expected product of 230 bp (Itoh et al. 2015). PCR conditions were 35 cycles of thermal denaturation at 95 °C for 30 s, annealing at 50 °C for 30 s, and elongation at 72 °C for 45 s. After PCR, bands were confirmed on a 1.2% agarose gel by electrophoresis, and then excised for sequencing by TA cloning using a Mighty TA-cloning kit (Takara Bio, Japan). The gene sequence was decoded using a model 3130 genetic analyzer (Applied Biosystems, USA) and analyzed using MEGA Ver. 7 software.

The 5′Balspec16S (5′-CGCATGTATGAAGAAGACCA-3′) and 3′Balspec 16S (5′-TTACCTATATAATTGTCGATACCA-3′) primer were also used for AHB strain sequencing, with an expected product of 1075 bp (Tavares et al. 2006).

## Results

### Separation of culture and *B. mandrillaris*

**Fig. 2:**
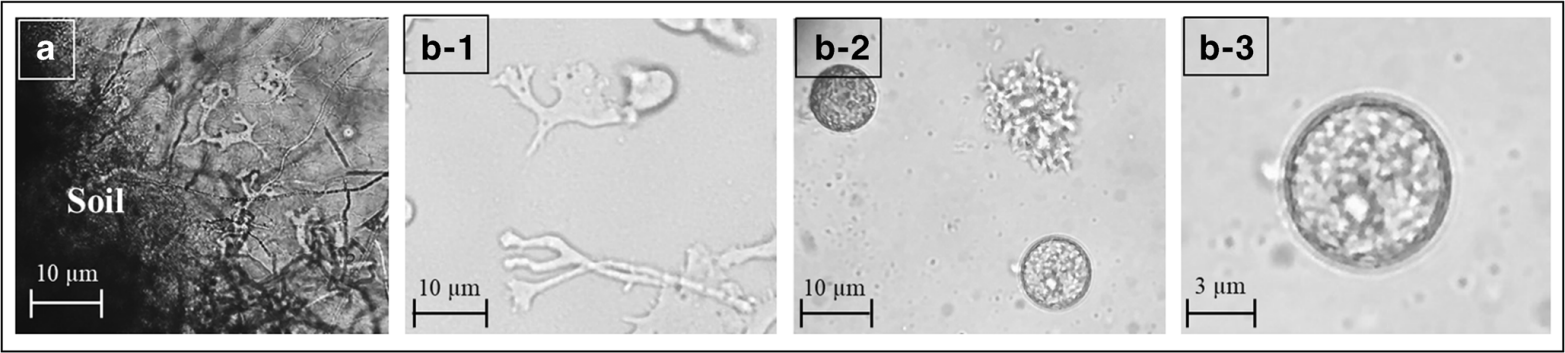
Morphology of amebae isolated from soil. (A) Trophozoites of large amoebae that appeared after 5 days of soil culture. (B-1) Trophozoites of AHB strain in SS medium. (B-2, B-3) AHB strain that formed cysts in SS

**Fig. 3:**
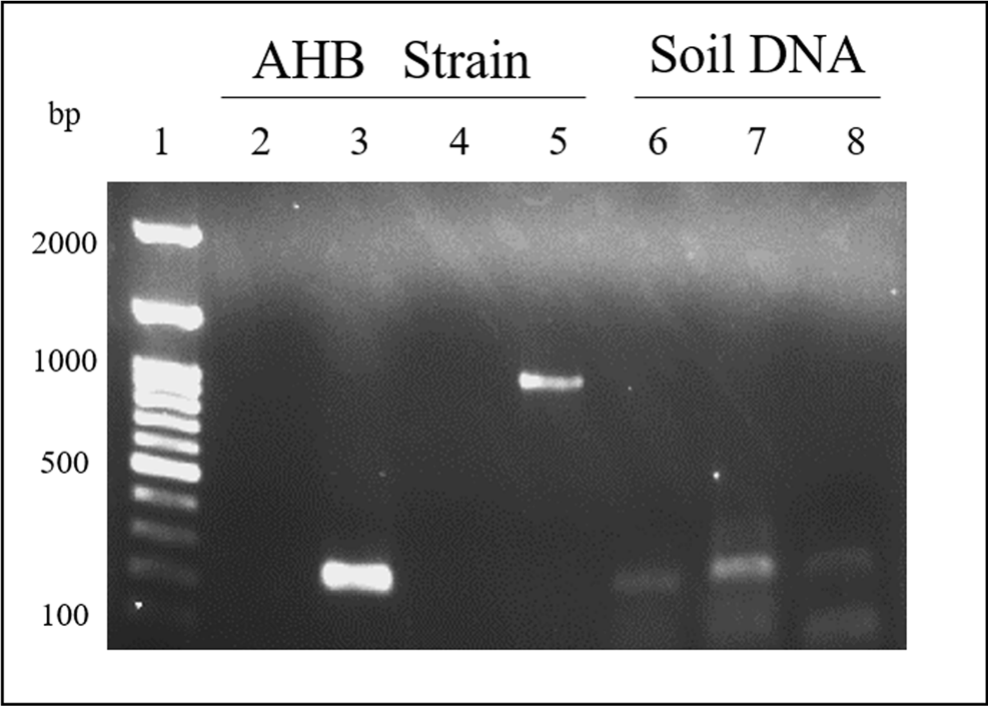
*Balamuthia*-specific-PCR for isolated amoebae and soil DNA. Lane (1) 100-bp ladder (Nippon Gene, Japan), lane (2) negative control, primer: 5′Balspec 16S and Balspec 16Sr 610, lane (3) sample of AHB strain, Primer: 5′Balspec 16S and Balspec 16Sr 610, lane (4) negative control, primer: 5′Balspec 16S and 3′Balspec 16S, lane (5) sample of AHB strain, primer: 5′Balspec 16S and 3′Balspec 16S, lanes (6, 7, 8) positive sample of soil DNA, primer: 5′Balspec 16S and Balspec 16Sr 610

Large dendritic amoebae were present in samples cultured from several sites after 5 days of culturing (Fig. 2(A)). Six types of dendritic large amoebae were isolated from six of 13 soil samples. The DNA extracted from the “amoeba No.1” sample, obtained from a horse farm in Hachinohe, was positive for *Balamuthia*-specific DNA (Fig. 3). This PCR product was used for sequencing after TA cloning and had 99% homology with the *Balamuthia* 16S rRNA sequence. The isolated amoeba was designated as the “AHB” strain (Aomori-Hachinohe-Balamuthia Strain). A longer portion (1075 bp) of the 16S gene was used to confirm the identity of this strain following PCR and TA cloning (Fig. 3), and the resulting product again shared 99% homology (Table 3) with the *B. mandrillaris* 16S gene (accession number: LC348995).

**Table 3:** AHB strain 1076-bp sequence homology

No.	Accession no.	Strain	Query cover	Ident	Source	Note/location	Reference
1	KT175741	*Balamuthia mandrillaris*: *V039*	100	99	3-year, 10-month-old pregnant mandrill	The San Diego Zoo Wild Animal Park/California, USA	Visvesvara et al. 1990
2	AF477017	*Balamuthia mandrillaris*: *V433*	100	99	20-year-old gelding Thoroughbred	Diagnosed with CDC/unknown	Kinde et al. 1998
3	KT030673	*Balamuthia mandrillaris*: *SAM*	100	99	3-year-old female human, brain	Diagnosed with CDC/California, USA	Schuster et al. 2003
4	KT030672	*Balamuthia mandrillaris*: *RP5*	100	99	Environmental sample	Associated with Sam/California, USA	Schuster et al. 2003
5	KT030671	*Balamuthia mandrillaris*: *OK1*	100	99	Environmental sample	Diagnosed with CDC/California, USA	Dunnebacke TH et al. 2004
6	KT175740	*Balamuthia mandrillaris*: *2046-1*	100	99	26-year-old human	Survivor case/California, USA	Alexander LG et al. 2015
7	AF477015	*Balamuthia mandrillaris*: *V416*	100	99	10-year-old female human, brain	NA/S. Brisbane, Australia	Booton et al. 2003
8	AF477014	*Balamuthia mandrillaris*: *V194*	100	99	60-year-old male human, brain	Chronic alcoholic, seizures, hemiparesis/Las Vegas, Nevada, USA	Booton et al. 2003
9	KT175738	*Balamuthia mandrillaris: V188*	100	99	60-year-old male human, brain	Amputation of right leg at knee/Georgia, USA	Visvesvara et al. 1990
10	KT175739	*Balamuthia mandrillaris: GAM-19*	100	99	V188-frozen stock	–	Alexander LG et al. 2015

*NA* data not available

The nutritional type of the AHB strain was 15 to 45 μm in size and the cyst was 5 to 15 μm, which is similar to the morphology of *B. mandrillaris* in the literature (Schuster et al. 2003; Niyyati et al. 2009) (Fig. 2(B-1), (B-2), (B-3)).

In order to subculture the six large amoebae isolated from the soil samples, we switched from plate culture to BM-3 liquid culture, which is a suitable medium for culturing *B. mandrillaris* (Schuster and Visvesvara 1996). However, the amoebae did not survive in liquid BM-3 culture, and we therefore created a new simplified medium. We designed a medium containing soil, as we hypothesized that it would best support amoebae isolated from the soil environment. To test the new medium, we cultivated a laboratory strain of *B. mandrillaris* of environmental origin. The transplanted amoebae temporarily differentiated and proliferated, but gradually weakened and died without forming cysts.

### *Balamuthia*-specific PCR using soil DNA

In the present study, 13 soil samples were cultivated to obtain six large amoebae cultures. These were separated from soil of sampling place No. 1, 4, 5, 7, 10, and 13. Among these, only one clone was successfully obtained (AHB strain) from soil of sampling place No. 1. For this reason, we extracted DNA from 13 soil samples and used the DNA to confirm the presence of *Balamuthia* by *Balamuthia*-specific-PCR. As a result, *Balamuthia*-specific PCR amplification products in three soil samples (No. 7, 8, 10) were confirmed (Fig. 3). Following sequencing and analysis of these PCR products, we found that these putative *Balamuthia* strains shared 99% homology with previously reported *B. mandrillaris*. The accession numbers of these three PCR products are LC349292, LC349293, and LC349294.

## Discussion

*B. mandrillaris* was first discovered in 1986 from cerebral necropsy of a mandrill baboon (*P. sphinx*) that died of neurological disease at the San Diego Wildlife Park in California (Rodríguez-Zaragoza 2008), and is thus a relatively recently discovered pathogen. Since then, more than 200 cases of meningoencephalitis caused by *B. mandrillaris* had been reported worldwide by 2008 (Visvesvara et al. 1990; Siddiqui and Khan 2008). Many of the infected people are Hispanic with agriculture-related occupations, which involve contact with the soil, and may have provided the opportunity for infection (Schuster and Visvesvara 2004; Jackson et al. 2014). Consistent with this, *B. mandrillaris* has been found in environmental samples since the 2000s. To date, eight environmental cases have been confirmed, comprising two cases isolated from the soil in flowerpots in the USA (Schuster et al. 2003; Yagi et al. 2005), two from Iran (one from soil and one from city dust) (Niyyati et al. 2009, 2016), one from well water in Guinea-Bissau (Baquero et al. 2014), one from Peruvian soil (Cabello-Vílchez et al. 2014), one from water in Mexico (Lares-Jiménez et al. 2014), and one from a mud bath in Jamaica (Todd et al. 2015). These cases originated in regions with tropical climates, desert climates, and step climates. Similarly, the cases of human infection are concentrated in warm climates (Schuster and Visvesvara 2004). However, in the present study, we isolated *B. mandrillaris* from a heavy snow area in Japan, suggesting that this pathogen can survive in a wider range of environments around the world.

In the present study, we did not characterize the pathogenicity of the AHB. Although we considered it necessary to investigate the pathogenicity of the isolated strain, and attempted mass culture using the devised SS liquid medium, the AHB Strain could not be successfully cultured in this medium. For this reason, although SS medium was specifically developed for mass culturing soil-derived *B. mandrillaris*, it did not enable long-term cultivation of this strain. Cultivation of environmental strains may not be successful using currently available culture-based methods. It is necessary to develop new methods, which are suitable for mass culture of environmental isolates.

## Conclusions

The habitat of *B. mandrillaris* may extend to a wider area than previously expected. *B. mandrillaris* infection has a strong association with immune dysfunction, and so is likely to occur in infants, middle-aged, and elderly people, and those with underlying diseases such as HIV (Perez and Bush 2007; Schuster and Visvesvara 2004). In Japan, where the proportion of elderly people is increasing, such infections may become more prevalent in the future. While *B. mandrillaris* is currently classified as an infectious disease of warm climates like tropical climates and tropical climates, we have shown that it can be isolated in cold regions with heavy snow and must therefore re-assess the risk profile of this infectious amoeba in such regions.
